# Commentary: Synthetic Ubiquinones Specifically Bind to Mitochondrial Voltage-Dependent Anion Channel 1 (VDAC1) in *Saccharomyces cerevisiae* Mitochondria

**DOI:** 10.3389/fmolb.2017.00016

**Published:** 2017-03-24

**Authors:** Manuel Gutiérrez-Aguilar

**Affiliations:** Department of Medicine, Washington University School of MedicineSt. Louis, MO, USA

**Keywords:** mitochondrial permeability transition pore, yeast mitochondria, VDAC, ubiquinone derivatives, mitochondrial uncoupling

The mitochondrial permeability transition pore (PTP) is an unselective channel that collapses the proton electrochemical gradient of inner mitochondrial membranes. Opening of the PTP results in ATP hydrolysis and is thought to mediate heart and brain injury after periods of ischemia-reperfusion (Biasutto et al., 2016). The PTP or PTP-like structures have been detected in mammals, plants, insects, and yeasts. Although some properties of the mitochondrial channel in each model organism may significantly differ from others (Bernardi et al., 2015; Gutiérrez-Aguilar and Uribe-Carvajal, 2015). Yet, PTP research has been largely hampered by the lack of a definitive molecular identity for this channel. That being said, several protein candidates thought to form part of this pore have not passed rigorous loss-of-function genetic approaches. This has been true for the inner membrane Adenine Nucleotide Translocator (ANT) and Phosphate Carrier as well as for the outer membrane Voltage Dependent Anion Channel (VDAC) (Kwong and Molkentin, 2015). However, the possibility that these (and other) proteins rather modulate the PTP through mitochondrial availability of adenine nucleotides, phosphate and other PTP effectors has remained an open question.

In a recent study, Murai and colleagues aim to detail the molecular mechanism by which ubiquinone analogs bind VDAC1 to prevent a Ca^2+^-induced, mitochondrial PTP in the yeast *Saccharomyces cerevisiae* (Murai et al., 2017). To achieve this, the authors synthesized specific ubiquinone derivatives (PUQ) to perform photoaffinity labeling of VDAC1, and thus studying the docking of these molecules to VDAC1 at the amino acid level.

The authors successfully labeled isolated yeast mitochondria with the derivatives PUQ-1 and PUQ-2 and consistently detected a ~30 kDa band when protein lysates were resolved on an SDS-PAGE setting. Moreover, PUQ binding to ~30 kDa proteins was also detected in more complex electrophoretic settings and shown to be counteracted by a non-photosensitive PUQ analog, suggesting the selective binding of PUQs to a discrete protein. Mass spectrometric characterization of the protein stained at ~30 kDa resulted in the detection of the yeast protein Por1, also known as VDAC1. These results were validated by means of VDAC1 purification and labeling using a well-established chromatographic method and by Western blotting of the PUQ-VDAC1 bound complexes. It is important to mention that PUQ labeling with classical ubiquinone-binding proteins such as SdhD (respiratory complex II subunit) or *Cyt b* (complex III subunit) was not detected, and PUQs were also shown to be poor mitochondrial substrates.

Importantly, Murai and collaborators showed that PUQ binding to VDAC1 was antagonized by more simple- and physiological-ubiquinones. The nature of these interactions was mapped by the authors using mass spectrometric approaches leading to conclude that the quinone heterocycle of the synthesized PUQs binds to a C-terminal domain clustered within Phe221 and Lys234. This domain presents positively charged residues present in both human and *S. cerevisiae* (Figure 1). Nonetheless, site-directed mutagenesis of potential hydrogen bond-forming residues resulted in no apparent changes in labeling. The authors suggested that the PUQ-VDAC interaction may not follow typical “lock and key” docking. To substantiate this proposal, PUQ-VDAC binding was assessed under different reducing conditions to modify hydrogen-bonds in the potential PUQ-binding domain. Under these circumstances, PUQ bound VDAC with the same characteristics.

**Figure 1:**
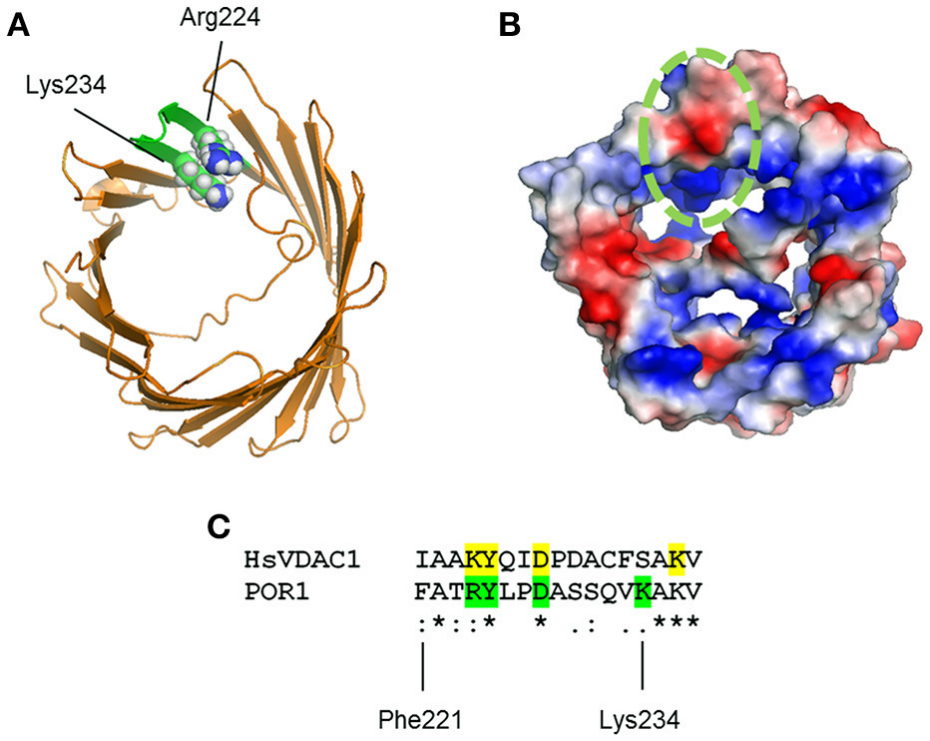
**(A)** Model of VDAC1 from PDB entry 2K4T showing amino acid substitutions to match *S. cerevisiae* VDAC1 (green). Arg224 and Lys234 are shown in sphere projection. **(B)** VDAC1 model in **(A)** is shown colored by qualitative electrostatic potential. The scale of the potential is ± 66.381 kBT/e. All surfaces were calculated using the protein contact potential function in PyMol. The potential PUQ-binding domain is circled in green. **(C)** Multiple sequence alignment of human VDAC1 (HsVDAC1) and *S. cerevisiae* VDAC1 (POR1) reveal partial similarity on the potential PUQ-binding domain. Residues mutated by Murai and colleagues on yeast VDAC1 are shown in green, whereas potential homolog residues in HsVDAC are highlighted in yellow.

In agreement with previous evidence (Gutiérrez-Aguilar et al., 2014), the authors showed that ubiquinone derivatives can decrease mitochondrial permeabilization, although under different experimental conditions. While this previous evidence showed a protective effect using the well-established PTP inhibitor decylubiquinone on the yeast mitochondrial unselective channel, Murai and colleagues measured the activity of different PUQs on the Ca^2+^-induced yeast PTP under the optimized experimental conditions reported by Yamada et al. (2009). These conditions require low phosphate levels plus the Ca^2+^ ionophore ETH129 to provide PTP-permissive Ca^2+^ levels in the mitochondrial matrix, since *S. cerevisiae* lacks a mitochondrial Ca^2+^ uniporter in the inner membrane (Kovács-Bogdán et al., 2014). It is worth mentioning that Murai and colleagues did not measure the effect of PUQs on the Ca^2+^-induced mitochondrial swelling (or respiration) in VDAC1 knockout yeasts. These key experiments would significantly test whether ubiquinones bind VDAC to regulate PTP opening, and validate previous studies showing VDAC as a regulatory component of the permeability transition in yeast (Gutiérrez-Aguilar et al., 2007).

Martin Crompton's pioneering work showed that cyclophilin D (CypD) could be used as a “bait” to pull-down protein complexes containing ANT and VDAC. However, the role of VDAC was then questioned by studies showing CypD could bind ANT but not VDAC in rat and yeast mitochondrial lysates (Woodfield et al., 1998). The role of VDAC was later addressed in studies showing that aminoketone derivatives were able to bind VDAC to suppress PTP opening (Cesura et al., 2003). These results were later disregarded by the same group by showing that these analogs were also able to bind a ~32 kDa protein in a VDAC1^−/−^ context and under conditions where no VDACs were interacting with their probe (Krauskopf et al., 2006). Further experiments by Molkentin's group showed that VDACs were dispensable for PTP opening and unlikely components of this pore (Baines et al., 2007). As mentioned above, this conclusion was also reached for *S. cerevisiae*, although VDAC1 was proposed to bind alkyl amines, polyanions, and even calcium to halt mitochondrial permeabilization (Gutiérrez-Aguilar et al., 2007). More recently, an RNAi-based high-throughput screen rendered VDAC1-silenced HEK293T cells more resistant to Ca^2+^-induced PTP (Shanmughapriya et al., 2015). Thus, the results presented by Murai and colleagues place VDAC1 as an obvious “gate” that may indirectly regulate the PTP (an inner membrane event) under certain experimental conditions. This proposal is also substantiated by recent reports showing that a new set of VDAC1 effectors reduce matrix Ca^2+^, oxidative stress and mitochondrial transmembrane potential dissipation in mammalian mitochondria (Ben-Hail et al., 2016). Consequently, VDAC1 targeting may still constitute a cogent strategy to control PTP opening and this possibility should be considered in more detail.

## Author contributions

The author confirms being the sole contributor of this work and approved it for publication.

### Conflict of interest statement

The author declares that the research was conducted in the absence of any commercial or financial relationships that could be construed as a potential conflict of interest.
